# The Role of Superabsorbent Polymers and Polymer Composites in Water Resource Treatment and Management

**DOI:** 10.3390/polym16162337

**Published:** 2024-08-18

**Authors:** Assunta Campanile, Barbara Liguori, Giuseppe Cesare Lama, Federica Recupido, Silvana Donatiello, Mariarita Gagliardi, Alfonso Morone, Letizia Verdolotti

**Affiliations:** 1 Applied Chemistry Labs-Department of Chemical, Materials and Industrial Engineering, University of Naples Federico II, 80138 Naples, Italy; assunta.campanile@unina.it; 2 Institute for Polymers, Composites and Biomaterials (IPCB-CNR), 80055 Portici, Italy; giuseppe.lama@ipcb.cnr.it (G.C.L.); federica.recupido@cnr.it (F.R.); 3 Department of Architecture (DIARC), University of Naples Federico II, 80134 Naples, Italy; silvana.donatiello@unina.it (S.D.); mariarita.gagliardi@unina.it (M.G.); alfonso.morone@unina.it (A.M.)

**Keywords:** superabsorbent polymers, water resource, urban water management

## Abstract

In the last century, the issue of “water reserves” has become a remarkably strategic topic in modern science and technology. In this context, water resource treatment and management systems are being developed in both agricultural and urban area scenarios. This can be achieved using superabsorbent polymers (SAPs), highly cross-linked hydrogels with three-dimensional, hydrophilic polymer structures capable of absorbing, swelling and retaining huge amounts of aqueous solutions. SAPs are able to respond to several external stimuli, such as temperature, pH, electric field, and solution composition and concentration. They can be used in many areas, from sensor technology to drug delivery, agriculture, firefighting applications, food, and the biomedical industry. In addition, new categories of functional SAP-based materials, mainly superabsorbent polymer composites, can also encapsulate fertilizers to efficiently provide the controlled release of both water and active compounds. Moreover, SAPs have great potential in wastewater treatment for the removal of harmful elements. In this respect, in the following review, the most promising and recent advances in the use of SAPs and composite SAPs as tools for the sustainable management and remediation of water resource are reviewed and discussed by identifying opportunities and drawbacks and highlighting new challenges and aims to inspire the research community.

## 1. Introduction

The greatest challenge for modern agriculture is to produce food in sufficient quantities and of sufficient quality to satisfy the world’s growing population, which is estimated to increase by 70–100% by 2050 [1]. The Food and Agriculture Organization of the United Nations estimates that the amount of water used in agricultural sector will increase by 14% by 2024. Therefore, the demand for agricultural water will also increase dramatically. In addition, water scarcity has become one of the main constraints undermining the human species’ survival and the nations’ progress [2].

Drought is a climatic condition that arises in both high- and low-rainfall areas and it mostly occurs due to precipitation reductions over an extended period of time, such as a season or a year [3]. Drought stress is a significant player in the agricultural ecosystem and can undermine food security worldwide [4].

On the other hand, rising human consumption leads to quick soil degradation and to a decrease in nutrient content [5]. The addition of soil improvers (e.g., animal manure, peat, lignite, and zeolite) is considered one of the best management practices with which to improve soil water retention capacity and thus protect plants from abiotic stress.

Another important issue is the sustainable management of urban areas. In fact, the high number of impermeable surfaces generates considerably more stormwater runoff than is generated in natural areas [6]. In fact, these areas are characterized by the well-known urban “heat island effect” [7] that significantly increases global warming [8]. These issues have been frequently addressed by employing innovative materials [9,10,11,12,13,14]. An alternative is represented by the creation of urban green areas, such as green roofs, and punctual urban furniture systems integrated with IoT (Internet of Things) monitoring systems [15,16,17]. This provides various additional benefits, such as air purification [18], increased thermal building insulation [19], significant energy savings [20], reduced noise [21], an enhanced aesthetic appreciation of the living and working space [22], reduced urban heat island effects [23], a habitat for a variety of organisms, and increased biodiversity [24]. When extending the green roof application field to adverse climates, especially considering that the thickness of the substrate (soil) is extremely thin, it is mandatory to design suitable irrigation tools [25,26,27,28,29,30,31,32,33]. 

Accordingly, an innovative water management practice, useful either in agriculture or in urban green area, can be the application of superabsorbent polymers (SAPs) as soil improvers and water retention tools.

**Figure 1:**
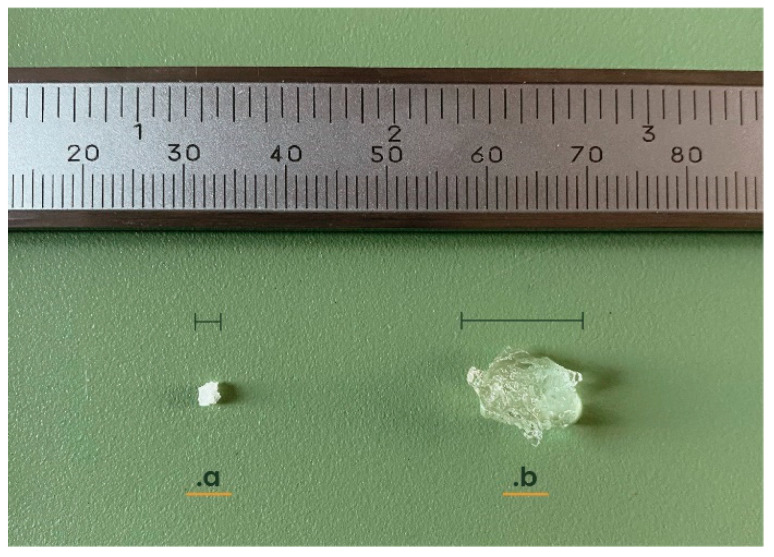
Dry (**a**) and hydrated (**b**) potassium polyacrylate hydrogel.

Superabsorbent polymers are hydrogels which are highly cross-linked with three-dimensional, hydrophilic polymeric structures able to absorb water, swell, and retain huge amounts of water or aqueous solutions [34,35,36,37], increasing their volume significantly, as shown in Figure 1. Specifically, hydrogels can retain around 10 g of water per gram of substance [38], while SAPs based on potassium polyacrylate hydrogels can absorb up to 1000 g [39].

**Figure 2:**
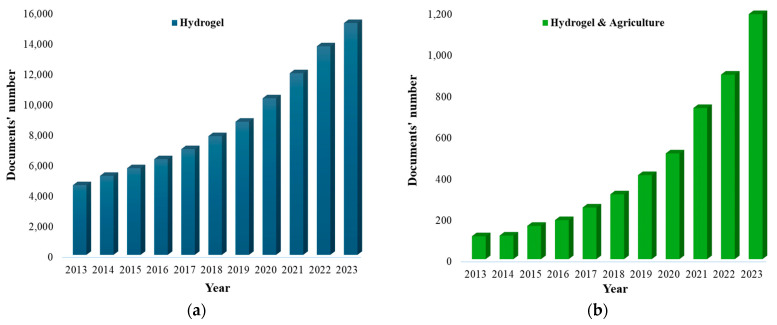
Number of publications on (**a**) hydrogel and (**b**) hydrogel use in agriculture over the last 10 years.

Analyzing the published literature from the past decade on hydrogels (SAPs) using the Scopus database, approximately 71,000 articles (Figure 2a) have been identified, showing a consistent upward trend.

On the other hand, when filtering with the keyword “agriculture”, a total of 4871 articles (5% of the total explored literature) are discovered concerning the use of hydrogels in agriculture (Figure 2b). This trend shows a rising interest in agricultural applications for hydrogels and SAPs.

The application of SAPs in agriculture emerged in the 1970s based on the idea of using these materials as soil amendments. Specifically, their first applications showed no beneficial effects and, in some cases, worsened normal plant growth [40,41,42,43,44]. Encouraging results were obtained by Hüttermann et al. in 1999, who proposed the application of a “new generation of soil conditioners” to ensure the survival of Slovenian *Pinus halepensis*, resulting in a significant increase in the plants’ drought tolerance [45].

SAPs can be also integrated within urban furniture systems, with the aim of achieving water resource management and smart irrigation systems in urban areas. Over the past several years, examples of building or urban planning interventions, aimed at enabling more sustainable water management, have been in place, predominantly in Northern Europe. The best known is Potsdamer Platz in Berlin [46]. The main objective of the project is above all the reuse of rainwater through three cisterns, with capacities of 2550 m^3^, that collect rainwater from the roofs of buildings in the area [47]. The roofs are 60% covered with green vegetation, so that rainwater is collected and purified and then reused for toilets or watering gardens. In addition, green roofs allow the temperatures of buildings to be lowered during summer seasons. In the context of green and smart urban furniture, Morone et al. presented an innovative approach based on the use of biofilters equipped with SAPs in combination with sensors inserted in modular industrialized elements to design a new generation of urban furniture [48].

In this review, the structure and properties of SAPs as water-retaining and controlled-release systems for fertilizers are highlighted. Challenges and opportunities in agriculture and urban green area scenarios are discussed, exploring the applications of advanced, multiperformance, and sustainable materials.

## 2. SAPs as Water-Retaining Systems

Water resource conservation is a global challenge, especially in times of climatic change. As water becomes increasingly scarce, the importance of water-retaining systems increases. Human survival, population distribution, urban development, land fertility, agricultural development and industrial progress depend completely on the water availability and sustainability of water-retaining systems [49]. 

Water-retaining systems involve invasive work on buildings, which is sometimes not feasible. From this perspective, using SAPs as materials for sustainable water-retaining systems can be an effective but non-invasive solution.

The ability of hydrogels to absorb water is due to the hydrophilic functional groups (such as hydroxyl (–OH), carboxyl (–COOH), amide (–CONH–), primary amide (–CONH_2_) and sulphonic (–SO_3_H) groups) bonded to the polymer backbone. These hydrophilic groups are essential for the cross-linking of polymer chains through physical or chemical bonds, leading to the formation of three-dimensional networked polymer structures [50,51]. The absorption mechanism is a combination of diffusive physical phenomena governed by Fick’s law between the chemical activity gradient inside and outside the polymer [52,53]. The properties of hydrogels depend on several important parameters such as hydrophilicity and degree of cross-linking of the polymer chains.

The main characteristics of hydrogels are a high adsorption capacity, regeneration capacity, and durability; photostability; non-toxicity; and a neutral pH [54,55]. Stimulus-responsive hydrogels can respond to environmental changes such as pH [56], temperature [57], electric field [58], solvent composition [59], and salt concentration [60]. Dual-responsive hydrogels can respond simultaneously to two external stimuli [61]. The essential characteristics of hydrogels, allowing their use in agriculture, are high water absorption and retention [62]. More generally, hydrogels have other specific characteristics, which are summarized in Table 1.

A variety of water-retaining agents (WRAs) are available on the market and they can be divided into three main categories according to their chemical nature [76]: synthetic polymers, natural-polymer-modified systems, and organic–inorganic composites.

### 2.1. Synthetic Polymers (SPs)

SPs are typically based on polyacrylic acid, polyvinyl alcohol, polyacrylamide, and polyethylene [77,78,79]. They have a high water absorption ratio, long shelf lives, and high gel strength. For the formation of hydrogels, one of several polymerization techniques can be employed, such as bulk polymerization, solution polymerization, and suspension polymerization. Overall, the monomer, the initiator, and the cross-linker are the three elements of the hydrogel synthesis reaction [80]. SPs have specific properties (high adsorption and regeneration capacity, external stimuli reactivity, and photostability) that allow their application in several fields. On the other hand, they have high costs and can hardly degrade, causing environmental pollution after many applications [81], thus limiting their application in agriculture and horticulture [79]. For this reason, attention has recently focused on the development of hydrogels from natural resources [82].

### 2.2. Natural-Polymer-Modified (NPM) Systems

NPM water retention systems were the first superabsorbent materials developed. The main natural sources used to produce NPMs are polysaccharides and polypeptides from animal collagen, plants, and seaweed. NPMs are often prepared by adding synthetic grafts (e.g., vinyl monomers) to natural substrates. For enhancing starch properties, the most suitable methods are chemical modification and graft copolymerization with vinyl monomers. Thus, the application fields of starch-based hydrogels can be further increased. The most used methods for the preparation of NPMs are generally based on graft copolymerization and biocatalytic reactions [80]. Natural hydrogels are characterized by low prices and high biocompatibility and the most widely used varieties are based on the modification of starch, cellulose, humic acids, lignin, amino acids, and chitosan [83,84,85,86,87,88].

### 2.3. Organic–Inorganic Composites (OICs)

There are three approaches used to improve hydrogel strength: increasing the cross-linking density [89]; surface cross-linking [90]; and the preparation of composite hydrogels by incorporating inorganic particles and/or specific polymers into the hydrogel [91,92,93]. Various types of mechanically stable hydrogels have been designed, such as interpenetrating network (IPN), semi-interpenetrating network (semi-IPN), double-network (DN), and nanocomposite (NC) hydrogels [94].

The synthesis of organic–inorganic composites (OICs) represents an interesting opportunity to improve the poor mechanical properties of blank hydrogels [95,96,97,98,99] and, at the same time, widen the functionality of the SAPs (i.e., as a fertilizer release controlled tool). Due to their hydrophilic nature, clays are very suitable as additives in absorbent materials. Additionally, various types of nanoscale materials, such as zeolites and carbon-based materials, are used for the preparation of nanocomposite hydrogels [100]. The recent scientific literature reveals the high performance of composite hydrogels as nutrient carriers; Ganguly and Das [101] synthesized a semi-IPN composite hydrogels for the controlled release of fertilizers by using nano phyllosilicate. The addition of the nano-clay increased both the mechanical strength of the gel and its water absorption capacity. Singh et al. [102] manufactured composites based on the use of hydrogel and zeolite previously loaded with phosphate as slow phosphate-releasing systems in the rhizosphere in acid soils, where low phosphate utilization efficiency due to the high rate of fixation is the main limitation in achieving higher productivity. Recently, a bio-based slow-release fertilizer, using superabsorbent polymers with natural char nanoparticles loaded with urea, has been studied to reduce nitrate leaching and improve the water retention capacity of soil, both at neutral and basic pHs [103].

## 3. SAP in Water Treatment

SAP hydrogels, thanks to three-dimensional ion networks, are able to adsorb several inorganic and organic pollutants from wastewater, with high adsorption capacities compared to conventional water adsorbents [104]. The chemical and porous structure of hydrogels can be properly designed based on the type of pollutant and, at the same time, it is possible to promote good recyclability for several cycles without affecting the adsorption capacity. Therefore, SAPs have been proposed in wastewater treatment for the removal of harmful elements, like organic dyes, toxic metals, and antibiotics [35,38,105]. The efficacy of hydrogels in water purification can be improved by adjusting their polymer structures with nanomaterials, leading to the creation of hydrogel nanocomposites [106]. The combination of nanomaterials and hydrogel networks, providing complementary surface functionalities, displays excellent technological potential in water treatment applications. 

The mechanical properties, swelling behavior, and recovery/reusability of hydrogels can be enhanced by incorporating organic and inorganic nanomaterials, including carbon-based substances such as graphene [107] and carbon nanotubes (CNTs) [108], metal oxides and MXenes [109,110], sulfides [111,112], magnetic materials [113], metals [114], nanoclays [115,116], zeolites [117], layered double hydroxides (LDHs) [118], metal–organic frameworks (MOFs) [119,120], and nanocellulose [121,122].

Naturally occurring polysaccharides like cellulose, chitosan, and alginate are frequently utilized to produce hydrogel nanocomposites owing to their excellent adsorption capabilities, rapid kinetics, and ability to be reused.

Carbonaceous (e.g., graphene, GO, and CNTs) hydrogels are the most extensively studied adsorbents for efficient water treatment due to their significant surface area, elevated porosity, distinctive surface characteristics, strong chemical stability, ease of modification, straightforward regeneration, and ability to be reused.

The addition of clay in hydrogel nanocomposites achieved encouraging results in the removal of heavy metal (Cu (II), Cd (II) and Pb (II)) [123] organic dyes [124] and Ciprofloxacin [125] from aqueous solutions.

Natural and synthetic zeolites were also used to produce nanocomposite hydrogels with high adsorption performances, either in color adsorption from the dye mixture [126,127] or heavy metal [117,128] removal from the wastewater.

Nevertheless, the costs associated with hydrogel nanocomposites represent a significant barrier to their commercialization for water treatment applications. Achieving the cost-effective large-scale production of hydrogel materials could lower the overall expenses involved in the process [124,125].

## 4. SAPs, Pristine and Composite, as a Controlled-Release System for Fertilizers

SAPs (pristine and composite) can be also used to encapsulate fertilizers and ensure their slow release into the soil. Nitrogen is an essential plant nutrient, and nitrogen fertilizers are the most widely used in agricultural applications. Due to its high nitrogen content (46%), urea is the most widely used. However, only a very small percentage of the applied urea is consumed by plants due to its high solubility in water. This results in the use of a very large amount of urea, which increases costs and also pollutes surface water and groundwater. Therefore, hydrogel use can be a solution to increase the efficiency of nitrogen uptake by plants and reduce serious environmental problems [129]. More generally, due to superficial drainage, leaching and evaporation, the plant uptake of various nutrients decreases. These losses can be reduced by natural controlled-release formulations (CFRs) of agrochemicals, such as fertilizers, pesticides, herbicides, microbicides, and plant growth regulators [83]. In addition to reducing nutrient losses, CFRs have many advantages such as a constant or controlled supply of nutrients over a long period of time, increased fertilizer efficiency, reduced application frequency, and fewer negative effects from overdose, toxicity, and environmental pollution [130].

Fertilizers can be incorporated into a hydrogel using two different procedures. The first consists of an in situ method, in which they can be added to the reaction mixture and polymerized at the same time as the polymer matrices [131]; during the second procedure, the dry gel is swelled in the fertilizer solution. Subsequently, the hydrogel is dried, and the device is obtained. Each technique has advantages and disadvantages: for the first method, the trapped compound can influence the polymerisation process and the structure of the polymer network; for the second method, the charged compound can accumulate on the surface during the drying of the charged hydrogel, decreasing water uptake [131]. Teodorescu et al. [132] prepared synthetic CFRs based on cross-linked poly(acrylic acid) hydrogels and liquid fertilizers. The monomer and initiator concentrations and the fertilizer and cross-linking agent compositions significantly influence the degree of swelling of hydrogels. Downstream of synthesis parameter optimization, high swelling capacities and slow-release properties are obtained in still-distilled water at room temperature, opening the way for interesting future developments. Liu et al. [133] synthesized a slow-release superabsorbent nitrogen fertilizer (SSNF) in the presence of urea. The results of the research indicated that an SSNF is a type of multifunctional water management material that can be applied in agriculture and the restoration of arid and desert environments. In this account, SSNF is characterized by its slow nitrogen release and soil moisture conservation properties at the same time. Increased surface cross-linking, which can be achieved by optimizing the synthesis conditions, can improve the slow release and water retention properties.

A novel composite hydrogel was formulated with polyacrylamide, methylcellulose, and calcic montmorillonite for the controlled release of urea [134]. The presence of montmorillonite ensures a slower release of fertilizer compared to the case of pristine hydrogel at different pH values. Furthermore, the nutrient desorption kinetics are significantly lower than those of pure urea, thus avoiding leaching losses.

An innovative synthesis process was presented by Rashidzadeh et al. [135]. The controlled-release device was prepared by coating commercial NPK fertilizer granules with a hydrogel/clinoptilolite nanocomposite. By combining the well-known properties of natural zeolites, such as clinoptilolite, in agricultural applications and the high-water retention capacity of hydrogels, excellent results have been achieved in terms of the controlled-release formulation of fertilizer.

## 5. SAPs in Urban Green Areas

**Figure 3:**
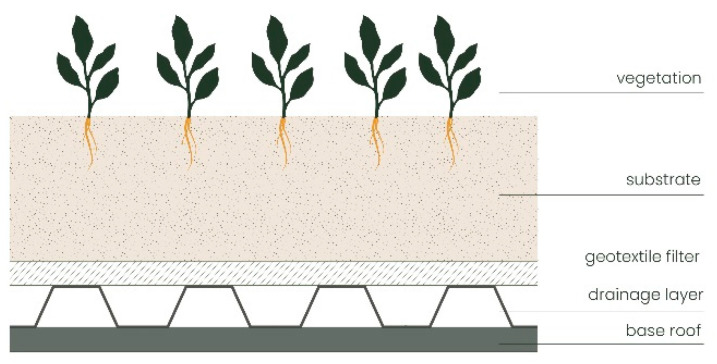
Experimental setup of the green roof, readapted from [137].

As regards recent scientific research into green roof substrates, [136,137,138,139] compared the water retention capacity of a traditional green roof substrate with three innovative ones that contained two different soil conditioners: (1) potassium polyacrylate hydrogel, administered in different weight percentages, and (2) expanded clay and perlite. By monitoring the water retention capacity of drainage elements and substrates without adding vegetation states, it can be concluded that hydrogel works better than perlite and expanded clay, but the use of an excessive amount can have a negative impact on the water retention capacity of green roofs [136]. *Salvia elegans*, with its bright red flowers and fragrant leaves, is widely used in green areas, but this species is particularly sensitive to drought. Therefore, the effects of different amounts of superabsorbent polymer (sodium polyacrylate) as a function of three substrate types on the growth of *Salvia elegans* on green roofs were analyzed [137]. The substrate was prepared by mixing coir dust and perlite in different ratios and the effect of the season and rain was also evaluated. The experimental setup (Figure 3) shows that the optimal substrate for the growth of *Salvia elegans* in green roofs is perlite during both the dry and rainy seasons with a hydrogel addition greater than 1.0 kg m^−3^.

Young et al. [138] evaluated the survival of two common green roof plants (*Festuca ovina* and *Linaria vulgaris*) during an extreme drought condition (25 days) using two types of soil conditioner at different particle sizes: polyacrylamide gel and red brick. Experimental analysis has suggested that, to increase the drought resistance of green roofs, it is possible to use the following:

(1) Polyacrylamide gel at a high application rate (1 vol%), providing a water reserve;
(2) Coarser particle sizes of red brick, promoting slower and more sustainable plant growth.

Ju et al. [139] evaluated the effects of different hydrogel concentrations in various green roof substrates on mint (*Mentha spicata*) growth related to the rain. The study optimized the effect of both the composition of the substrate, in terms of the amount of perlite and coir, and the effect of hydrogel (sodium polyacrylate) on the survival of the mint plant during the dry period. The results show that the addition of hydrogels in green roofs increases the variety of plants that can be used in dry climates due to the improved water retention of the substrate.

Again, in the above-mentioned applications, SAPs were used, mixing the powder with the soil directly. A new sustainable approach has been used in the context of NBSs placed within the urban context, for example, during the urban green and smart furniture project AURA, in which the controlled-release system of water and nutrients through SAPs was applied.

**Figure 4:**
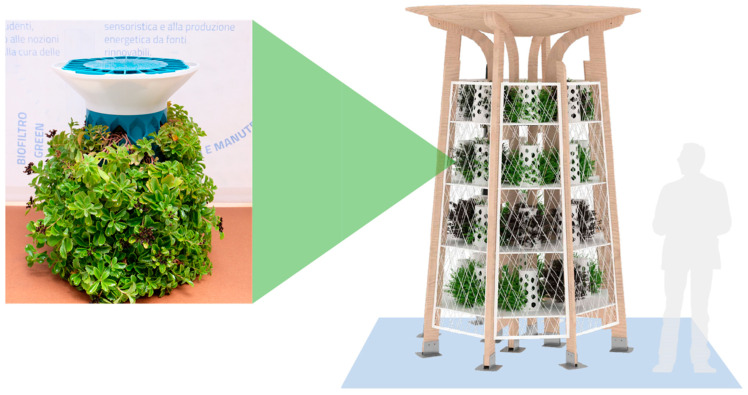
Urban green and smart furniture designed during AURA project [48].

AURA research developed a new generation of environmentally friendly urban furniture that, in addition to its original function, features bio-absorbent vegetation, referring to atmospheric pollutants, and is smart, as it is equipped with IoT-based intelligent sensors, being able to transmit climate data on urban pollution and vehicular traffic, plus other utility devices [48]. The systems involve the installation of a basic module consisting of a plant biofilter (Figure 4). This can be repeated and scaled up.

With this premise, different types of urban furniture have been designed, such as a drop (canopy), spot (pole), green wall, and totem system. The biofilter, made by additive manufacturing technology, contains soil for plant growth and a central canal where SAPs are located. The presence of SAPs ensures that water is released gradually according to the water needs of the plants. The material acts as a tank for water and nutrients and allows plants to avoid drought stress, reducing the systems’ maintenance cycles. In IoT monitoring systems, the health of the system is monitored and the need for the biofilter to be irrigated is indicated. In this way, it is possible to have the controlled and conscious management of water resources.

The nature-based approach in the AURA project, therefore, is not limited to the use of plants as a natural element for air filtering and monitoring, but is also implemented as a model for system running and structuring.

## 6. How to Use WRA and Long-Term Stability Assessment

As mentioned above, the SAP is a water reservoir within the soil, which is discharged at the root’s demand through the osmotic pressure difference.

**Figure 5:**
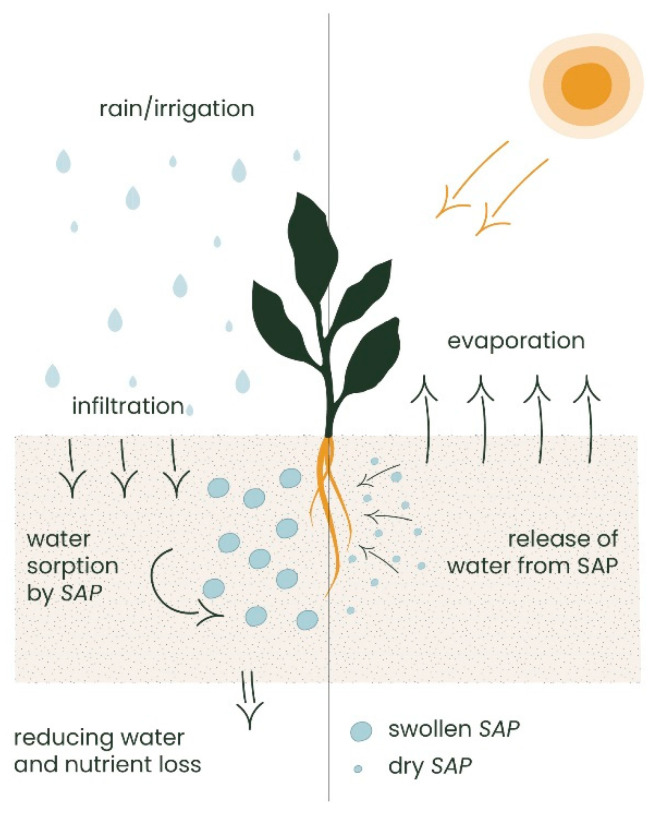
SAP operating mechanism as a soil conditioner, readapted from [140].

During rainfall and/or irrigation, SAP absorbs and retains an enormous amount of water and acts as an additional water reservoir (Figure 5). When the soil dries out, the water absorbed by the SAP is released into the soil [140]. In addition, it retains drainage water, recovering the amount of water that is normally lost through percolation and reducing the frequency of irrigation. Therefore, the traditional use of WRA provides the direct addition of SAP into the soil. As the granules absorb water, they swell and expand by increasing the soil porosity and providing an improved oxygen supply to the roots. In this way, it increases soil permeability and improves germination rates. Unfortunately, this practice hinders the possibility to regenerate/recover the sorbent.

Table 2 reports several examples of SAPs (synthetic polymer, SPs; natural-polymer-modified, NPM; and organic–inorganic composites, OICs) being used as soil improvers. Five parameters were highlighted: SAP chemical nature, absorption capacity, SAP/soil ratio and contact time, and regeneration. Specifically, regeneration refers to the hydrogels’ ability to re-absorb water while being applied in the soil. Its efficacy is evaluated over a longer or shorter time period, depending on the specific plant used.

In particular, Tan et al. [141] synthesised a natural SAP from food waste as a soil supplement. By applying the SAP in different amounts within the soil, it was shown that it improved both plant growth and irrigation water use efficiency. In particular, the yield of vegetable cultivation was increased by 88% and 113% by adding 3 and 5 wt% of SAP, respectively. In addition, increasing the frequency of irrigation improved plant growth in soil containing SAP because nutrient leaching was also inhibited. The application of a synthetic SAP, Stockosorb^®^ hydrogel, to olive crop under rain conditions increases soil water content and tree water status by improving the final productivity of olive trees [142]. The nanocomposite SAPs, prepared by Songara et al. [143] using natural materials (zeolite and guar gum), were tested in a real system (pot of rose).

These promising results enable the application of these composite SAPs as nutrient carriers and soil conditioning agents, especially in arid and semi-arid regions, due to their high potassium concentrations and water uptake rates.

**Table 2:** SAPs as soil improvers (* = swelling in distilled water).

Type of SAPs		Swelling in Tap Water, %	SAP to Soil Ratio, wt.%	Application Time, Day	Regeneration	Ref
**SP**		150	400 g/tree	Up to 360	Rain	[142]
		185	0.01	Up to 35	–	[78]
		–	0.5	Up to 15	–	[144]
		96.7–122.1	0.1–0.3	Up to 28	4 times (by adding water)	[145]
		–	0.04–0.4	Up to 77	8 times (by adding 1.2 l of water for plant)	[45]
		350 * (T = 50 °C)	0.1–0.2	Up to 22	–	[65]
		604 *	0.01	Up to 7	–	[66]
		–	2.5 kg/ha	Up to 84	12 times (by adding water)	[69]
		190	0.006	Up to 22	–	[70]
		–	0.1–1	Up to 30	–	[71]
		1665.8 *	0.002	Up to 270	–	[72]
		300–350	250 g/m 25 g/tree	Up to 90 Up to 360	Rain	[81]
		180	0.01	Up to 30	–	
**NPM**		350–800 * (T = 50 °C)	2.5–5 kg/ha	Up to 365	Irrigation and Rain	[73]
		187	3–5	Up to 30	4–10 times (by adding water or Hoagland’s solution)	[141]
		390 *	0.01	Up to 28	4 times (by adding water)	[85]
		1107 *	0.001–0.01	Up to 28	–	[88]
**OIC**	tuff	22.24–34	0.01	Up to 30	–	[135]
	nano char	215.1 *	1	Up to 7	–	[103]
	zeolite	92.5	–	Up to 16	2 times (by adding water)	[70]

## 7. Conclusions and Perspectives

Water is one of the most important components in plant growth and a water deficit during severe drought causes a substantial decline in crop yield. Therefore, superabsorbent polymers (SAPs) are excellent candidates for increasing plant growth by reducing water requirements and preventing water leaching. SAPs can retain and release high amounts of water and nutrients too, being part of slow-release smart fertilizers. According to the recent literature, hydrogels can be applied as water reservoirs and nutrient carriers to improve soil performance, even under extreme environmental conditions. Additional features are related to the high ability of hydrogels to adsorb several inorganic and organic pollutants from wastewater, which can be improved by using hydrogel nanocomposites. They also represent a possible solution that can be added to green roofs and urban furniture to significantly reduce stormwater runoff in cities. Despite water shortages, the employment of hydrogels in irrigation is not yet well recognized. As a result, potential arises for the development of sustainable hydrogels with bio-based raw materials that are naturally biodegradable and thus protect the environment and soil fertility, as synthetic SAPs, derived from petroleum resources, can potentially be harmful to the soil. Accordingly, future research activity will focus on a way to ensure the hydrogel recovery and regeneration. In addition, future research could focus on the development of composite hydrogels that exhibit higher mechanical properties and, by using soil improvers already widely used in agriculture as fillers, e.g., natural zeolites, also show high nutrient retention and release capacities, preventing nutrient leaching and bioaccumulation.

There is a need to find sustainable solutions for the agriculture industry that are closer to the aims of a greener and circular economy. For this reason, exploring applications of advanced, multiperformance, and sustainable materials, such as composite multiperformance hydrogels, is one of the most effective answers, pushing towards sustainable industrial growth.

Finally, a number of important milestones, listed below, remain open:

- Developing new types of green composite hydrogels from sustainable raw materials (such as starch and cellulose) with multi-performance properties, including water retention capacity and the controlled release of water and/or fertilizers, is important. The non-optimized use of fertilizers remains a major factor in environmental pollution. Encapsulating fertilizer granules in superabsorbent polymer (SAP) systems, traditionally used only as water reservoirs, can reduce overdosing and minimize the frequency of fertilizer application. Specifically, incorporating capsule-like fillers (such as zeolites, diatomite, clays, etc.) into hydrogels can enhance their functionality as nutrient/fertilizer reservoirs. These capsules facilitate controlled nutrient release, allowing crops to absorb nutrients efficiently and thereby reducing soil and water pollution.
- The development of sustainable smart hydrogels focuses on creating materials that are stimuli-responsive, i.e., capable of reacting to various environmental factors such as pH, temperature, moisture levels, and light. These advanced hydrogels can be particularly beneficial in agricultural applications, where they facilitate the controlled release of water and nutrients, enhancing crop growth and resource efficiency. These developments highlight the potential of green composite hydrogels in creating more sustainable and efficient agricultural practices.
- Developing new sustainable and regenerable composite hydrogels that can be easily regenerated after fulfilling their role in releasing water and/or nutrients remains a significant challenge. This endeavor is crucial within the framework of the circular economy. This challenge can be addressed by integrating Covalent Adaptable Networks (CANs) into hydrogels’ chemical structures. CANs are characterized by their ability to form and break covalent bonds reversibly, allowing the material to be repaired, reprocessed, and recycled.

Finally, the development of sustainable, high-performance, smart, and recyclable composite hydrogels with tailored, multifunctional properties remains a crucial topic. But these advanced materials have the potential to meet market and consumer needs, replace traditional products, and accelerate the green revolution towards sustainable industrial growth.

## Figures and Tables

**Table 1:** Characteristics of hydrogels.

Hydrogels’ Characteristics	Reference
Greater resistance in a saline environment	[63]
Maximum absorption capacity at T = 40–50 °C *	[64]
Gradual release of absorbed water	[65]
High stability in the soil for at least one year	[66]
Reduction in herbicide and fertilizer leaching	[67]
Improved physical properties of soils, root growth, and density	[68]
Increased seed germination rate and seedling emergence	[69]
Reduced irrigation frequency and plant water stress	[70]
Delay of permanent wilting point	[71]
Cost-effectiveness	[72]
Biodegradability without formation of toxic species	[73]
pH neutrality after swelling in water	[74]
Photostability	[75]

* Characteristic temperatures of semi-arid and arid soils.

## Data Availability

Dataset available on request from the authors.
